# Health workers’ experiences, barriers, preferences and motivating factors in using mHealth forms in Ethiopia

**DOI:** 10.1186/1478-4491-13-2

**Published:** 2015-01-15

**Authors:** Araya Abrha Medhanyie, Alex Little, Henock Yebyo, Mark Spigt, Kidane Tadesse, Roman Blanco, Geert-Jan Dinant

**Affiliations:** Department of Public Health, College of Health Sciences, Mekelle University, PO Box 1871, Mekelle, Ethiopia; Department of Family Medicine, CAPHRI, School for Public Health and Primary Care, Maastricht University, PO Box 616, 6200 MD Maastricht, Netherlands; Department of Surgery, School of Medicine, University of Alcala, 28871 Alcala de Henares, Madrid, Spain; General Practice Research Unit, Department of Community Medicine, The Arctic University of Norway, Tromsø, Norway; Digital Campus, 21 North Drive, Littletown, Winchester, Winchester, S022 6QA England, UK

**Keywords:** Community health workers, Health extension workers, Midwives, Primary health care, Maternal health care, Mobile health, mHealth, Electronic forms, Smartphones, Mobile technologies

## Abstract

**Background:**

Mobile health (mHealth) applications, such as innovative electronic forms on smartphones, could potentially improve the performance of health care workers and health systems in developing countries. However, contextual evidence on health workers’ barriers and motivating factors that may influence large-scale implementation of such interfaces for health care delivery is scarce.

**Methods:**

A pretested semistructured questionnaire was used to assess health workers’ experiences, barriers, preferences, and motivating factors in using mobile health forms on smartphones in the context of maternal health care in Ethiopia. Twenty-five health extension workers (HEWs) and midwives, working in 13 primary health care facilities in Tigray region, Ethiopia, participated in this study.

**Results:**

Over a 6-month period, a total of 2,893 electronic health records of 1,122 women were submitted to a central computer through the Internet. Sixteen (69.6%) workers believed the forms were good reminders on what to do and what questions needed to be asked. Twelve (52.2%) workers said electronic forms were comprehensive and 9 (39.1%) workers saw electronic forms as learning tools. All workers preferred unrestricted use of the smartphones and believed it helped them adapt to the smartphones and electronic forms for work purposes. With regards to language preference, 18 (78.3%) preferred using the local language (Tigrinya) version of the forms to English. Indentified barriers for not using electronic forms consistently include challenges related to electronic forms (for example, problem with username and password setting as reported by 5 (21.7%), smartphones (for example, smartphone froze or locked up as reported by 9 (39.1%) and health system (for example, frequent movement of health workers as reported by 19 (82.6%)).

**Conclusions:**

Both HEWs and midwives found the electronic forms on smartphones useful for their day-to-day maternal health care services delivery. However, sustainable use and implementation of such work tools at scale would be daunting without providing technical support to health workers, securing mobile network airtime and improving key functions of the larger health system.

**Electronic supplementary material:**

The online version of this article (doi:10.1186/1478-4491-13-2) contains supplementary material, which is available to authorized users.

## Introduction

With the aim of ensuring access to basic promotive, preventive, curative and rehabilitative health services, many developing countries, including Ethiopia, have been revitalizing and accelerating the expansion of primary health care [1].

Since 2003, Ethiopia has been expanding access to primary health care through its community-based health extension programme (HEP) and primary referral health centres. Between 2003 and 2010, a total of approximately 34,000 health extension workers (HEWs) have been trained and deployed in approximately 15,000 newly constructed health posts. One health post was constructed for each of the 15,000 *kebeles* (villages) in the country. A *kebele* is an administrative unit synonymous with a village of approximately 5,000 people. The HEP is a package of seventeen components comprising four major programme areas: Family Health Services, Disease Prevention and Control, Hygiene and Environmental Sanitation, and Education and Communication. Within the Family Health programme area, HEWs are trained on how to provide and educate people within their *kebele* on maternal health care.

The acceleration of access to primary health care in Ethiopia has not only resulted in a significant increase in the number of health centres, but also in a remarkable increment in trained and deployed midlevel health professionals at health centres. The number of operational health centres in the country has increased by 413% from 519 in 2004 to 2,660 in 2011. Between 2004 and 2011, the number of deployed health officers increased from 683 to 3,702; midwives from 1,274 to 2,416; and all nurses (including midwives) from 15,544 to 29,550 [2–7].

Although there is a need for rigorous and systematic evaluation of the impact of this acceleration and expansion of primary health care in Ethiopia, improvements in maternal and child health care indicators over the past few years are highly likely attributed to this extensive and aggressive expansion [6, 8, 9]. Between 2005 and 2011, the contraceptive prevalence rate (CPR) increased from 15 to 29%, antenatal care (ANC) coverage increased from 28 to 44%, while infant and under-5 mortality declined from 77 and 123 deaths per 1,000 live births, to 59 and 88 deaths per 1,000 live births, respectively [10, 11].

Despite these achievements, the maternal mortality ratio remained the same: 673 per 100,000 live births in 2005 and 676 per 100,000 live births in 2011. In a similar time frame, the percentage of pregnant women who were assisted for birth by skilled birth attendants increased slightly (from 6 to 10%), as did those who gave birth at health institutions (from 4 to 10%) and those who received postnatal care (PNC) within first 2 days of delivery (from 5 to 7%) [10, 11].

Previous studies published on the health extension programme and primary health care in Ethiopia showed that the quality of maternal health care services was poor. These studies indicated the HEWs’ 1-year training might be inadequate and HEWs had poor knowledge and skills on maternal health care, and referral linkage between health posts and health centres was weak. Other reasons mentioned by these studies include workload, lack of motivation and incentives, culture, and the low health-seeking behaviour of the community. Moreover, these studies showed that the health information reporting system at primary health care settings was poor. Thus, national figures on key maternal health care indicators extracted from primary health care service reports might be highly subjected to errors [6, 12–15].

With the recent advent of multifunctional smartphone technologies and rapid penetration of mobile phone networks in developing countries, mobile health (mHealth) applications are widely perceived as potential solutions for addressing the needs and challenges of health workers and health systems [16–19]. The World Health Organization (WHO) defines mHealth as 'medical and public health practice supported by mobile devices, such as mobile phones, smartphones, patient monitoring devices, personal digital assistants (PDAs), and other wireless devices'. mHealth applications and programmes make use of several aspects of mobile technology such as text messaging, voice and video services and Internet connection [18, 19].

A framework for mHealth in Ethiopia issued in 2011 suggested mobile technologies can be used to address HEWs’ need of referral, training and education, supply chain management, data exchange and consultation [20]. In relation to reducing maternal mortality and improving maternal health, mHealth might have significant importance to bridge the gap between skilled birth attendants and community health workers, because mHealth applications could allow exchange of information. In addition, well designed electronic forms downloadable to smartphones for ANC care, delivery and PNC, could assist community health workers to easily identify danger signs and complications in pregnancy and thereby facilitate timely referral [16, 19–22].

Systematic reviews showed that many of the existing mHealth studies are conducted in the developed world and most of these studies dealt with the role of short message services (SMS) and voice call reminders. mHealth studies targeted on mHealth applications making use of electronic forms and Internet functionality of mobile technologies for health workers in developing countries are scarce [19, 21, 23]. Contextual evidence regarding barriers and facilitators in using electronic forms on smartphones by health workers in developing countries for maternal health care in day-to-day health care delivery is scant. Thus, introduction of health data collection using mobile applications in day-to-day health care delivery might encounter unforeseen challenges and resistance [16, 20, 24].

With the aim of introducing electronic forms on smartphones for data exchange and transfer, and assessment of pregnant women in day-to-day health care delivery at primary health care settings in Ethiopia, this study assessed quantitatively health workers’ experiences, barriers, preferences and motivating factors in using mobile health forms on smartphones in the context of maternal health care in Ethiopia. Moreover, this paper concludes by shedding light on the strategies and lessons learned for improving the use of such mHealth applications at primary health care settings.

## Methods

### Study setting

This study was done among midwives and HEWs. Midwives and HEWs are primarily responsible for the provision of maternal health care services at primary health care units (PHCUs) in Ethiopia.

**Figure 1:**
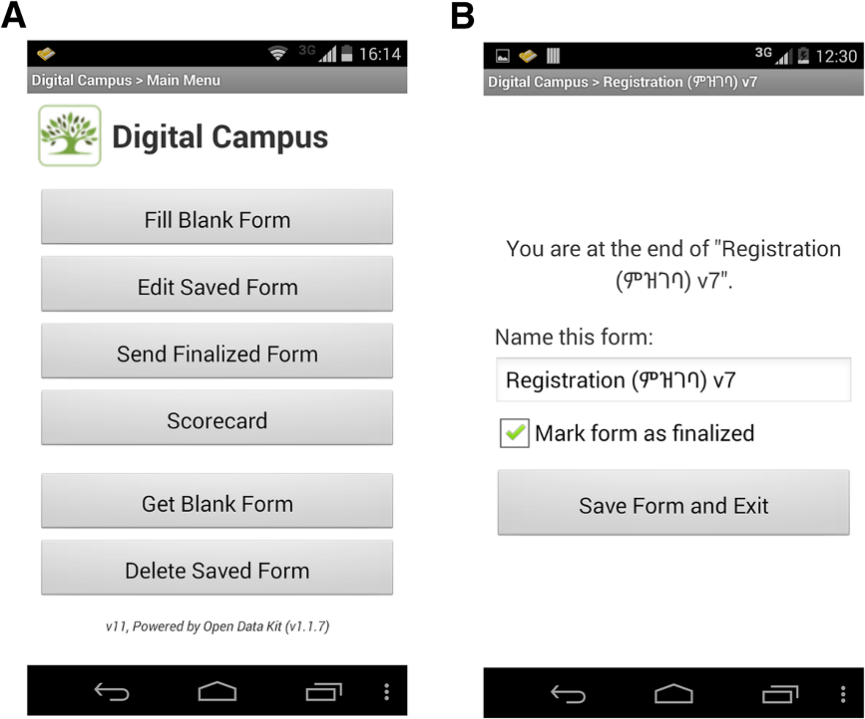
**Screenshots of Open Data Kit (ODK) home page (A) and ODK saving page (B).**

**Figure 2:**
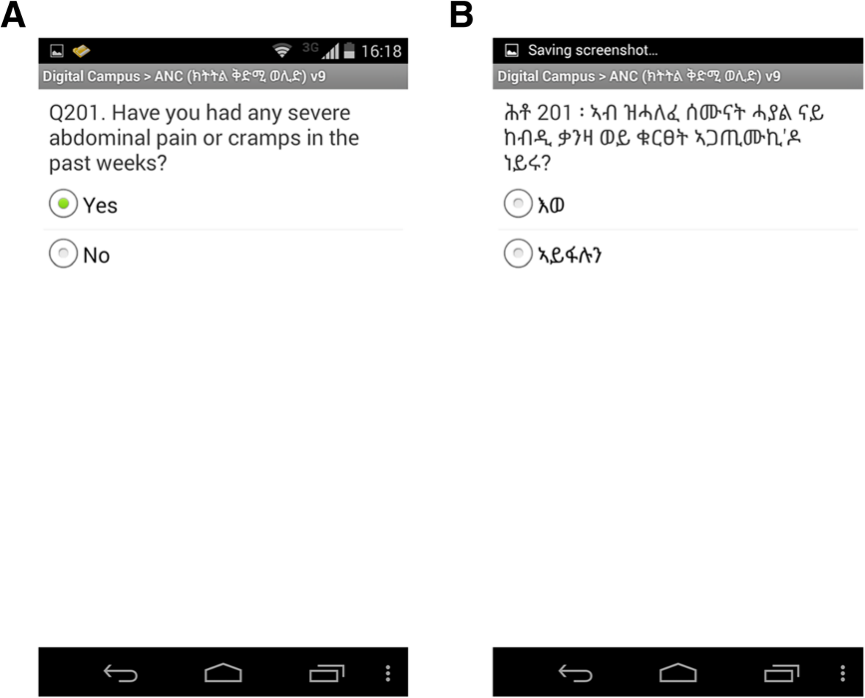
**Screenshots of sample question in English (A) and sample question in local language (Tigriyna) (B).**

**Figure 3:**
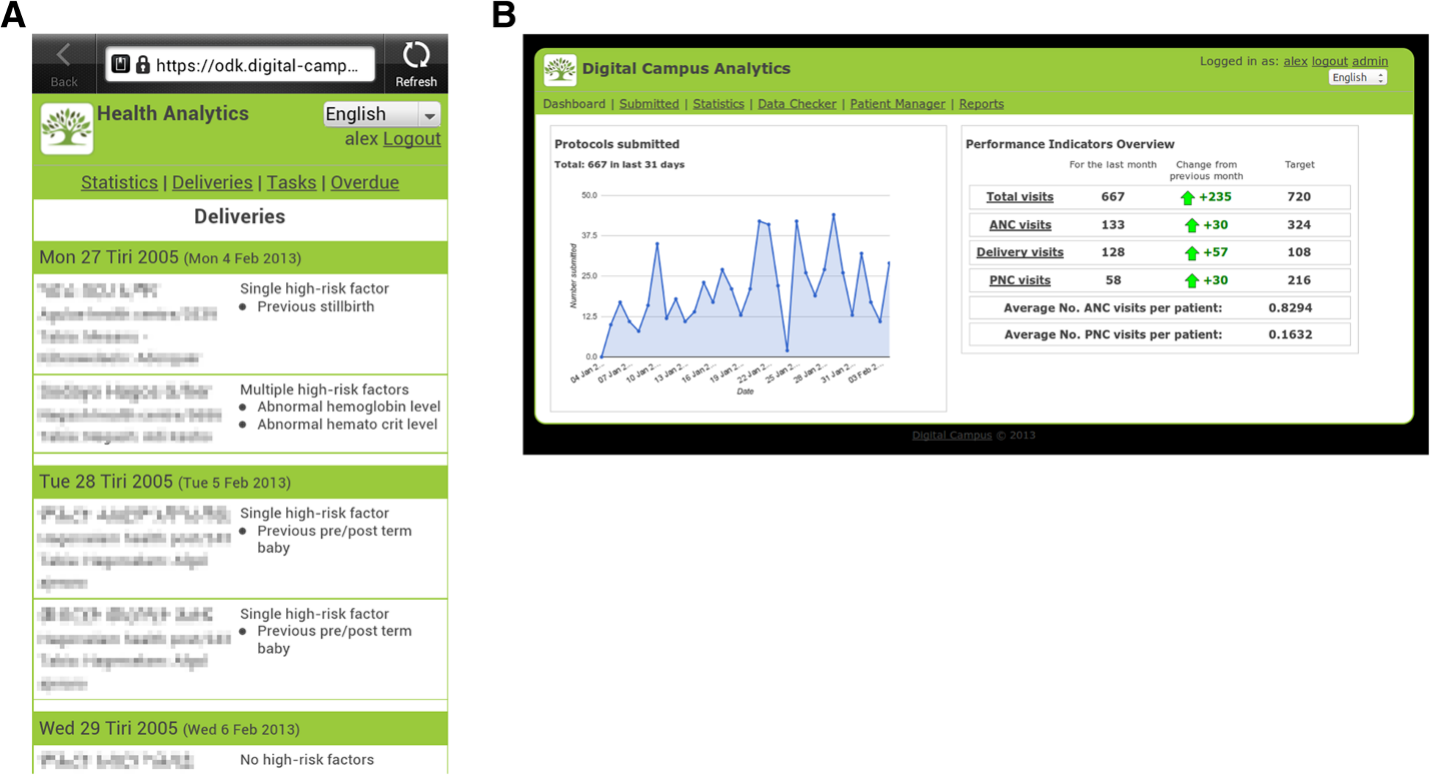
**Screenshots of mobile scorecard and analytics dashboard (A and B). Personal data has been pixilated (A).**

Technical details of the mHealth application, and electronic maternal health care forms employed in this study are described in another published article [25]. The technical components of the mHealth application developed and deployed as part of this study cover: 1) maternal health care forms; 2) scorecard/analytics dashboard. These components have been built on systems already available, using open source components. Figures 1, 2 and 3 show screenshots and figures that illustrate the application, sample questions in a form, scorecard and analytics dashboard.

#### Electronic maternal health care forms

We used a slightly customized version of Open Data Kit (ODK) open source software for the development of the forms (http://opendatakit.org/). Several customizations were made to make suitable use of the phones and ODK by the health workers in Ethiopia. These include: a) local language support; b) supporting local calendar and c) ODK widgets.

The forms consisted of a list of questions to be asked for mothers coming to the health post or health centre for maternal health services. We adopted the questions in the forms from the paper forms of the Ethiopian Federal Ministry of Health for maternal health services provision protocol at PHCUs. We created separate forms for patient registration, ANC, ANC laboratory tests, delivery care, PNC, pregnancy termination, and transfer forms. However, almost none of the health workers used the pregnancy termination and transfer forms. Hence, we excluded the analysis of these forms from this paper. The questions incorporated in the forms required different types of responses including: yes/no, multiple options, only one option, text, number, date, and Global Positioning System (GPS) coordinates.

#### Scorecard and analytics dashboard

We developed an analytics dashboard and a mobile scorecard to allow HEWs, midwives, their supervisors and the local health offices to track the progress of pregnant mothers, their medical and pregnancy risk factors, and a range of key performance indicators. Providing information back to health workers and their supervisors about their performance was designed to help the health workers manage their workload and patients. Performance indicators included the number of ANC, delivery and PNC visits made.

#### Workflow

Health workers used the electronic maternal health care forms downloaded on the HTC hero android smartphones for assessing and interviewing pregnant women. To do this every time a woman attended, the health worker had to launch the ODK software and open the needed forms. We did not replace the paper forms at health facilities with electronic forms. Rather we allowed the health workers to use the electronic forms in addition to the paper forms. Once a health worker had finished assessing a woman and completed a form, it was submitted to the server.

Training of the health workers and deployment of the mHealth application and forms began in August 2011 and the whole mHealth study extended until May 2013. However, this study assessed quantitatively the health workers’ experiences, barriers, preferences, and motivating factors in using the mobile forms for the period of 6- months (October 2012 to March 2013). During these 6 months, a total of 25 health workers (10 midwives and 15 HEWs) had been working in 9 health posts and 4 health centres in the selected 2 districts (Kilte Awlaelo and Hintalo Wajerat) of Tigray Region, Ethiopia. All these 25 health workers participated in the study. Over the 6-month period, almost all the health workers had been submitting real patient records to our central server using the electronic forms and smartphones. The numbers of records submitted by each health worker varied from zero to 372. Of the 25 workers, 2 did not submit any record. One was negligent to use the mobile forms at all while the other had difficulty in writing and reading the smartphone. Hence for this study, we mainly considered the activities and records submitted by the 23 health workers who did contribute to our server over the 6-month period.

### Data collection

Using a semistructured questionnaire (Additional file 1), we collected data from all health workers who submitted at least one electronic patient record during the study period. The questionnaire comprised questions related to sociodemographic characteristics of the health worker, prior use of mobile phone, motivating factors, preference, barriers, and satisfaction in using the electronic forms and smartphones for patient assessment. We developed and adapted this questionnaire based on the lessons we learned from pretest and feasibility assessments conducted prior to this study and literature review [26]. For this assessment, we chose to use a paper questionnaire instead of an electronic questionnaire, as there were several open questions in the questionnaire. In addition to the questionnaire, we collected health workers’ monthly use of mobile top-up vouchers (or mobile voucher cards) for voice calls, Internet and SMS services from the service provider, EthioTelcom, to analyze trends in health workers’ use of voice calls, mobile Internet and SMS during the study period. Mobile top-up voucher in this study refers to the amount of money required for buying a fixed airtime from the service provider. Data collection was completed by members of the research team (AAM and KT) who were fluent in the local language, Tigrinya.

### Data analysis

Data entry and analysis was conducted using SPSS version 16 (SPSS, Chicago, IL, USA). Responses to open-ended questions of the questionnaire were categorized and coded before entry. Coding was done by both research members who were involved in the data collection. Difference in coding was solved by consensus. We used descriptive statistics to describe health workers’ experiences, barriers, preferences and motivating factors in terms of frequencies and percentages.

### Ethical consideration

This study was approved by the Health Research and Ethics Review Committee of the College of Health Sciences of Mekelle University (number: ERC 0032/2011). Written consent for participation was obtained for each health worker. The health workers were informed about their right to withdraw from the study at any stage.

## Results

### Sociodemographic characteristics of study participants

The mean age of the health workers was 31 years (SD = 7 years), with 18 workers (72%) under 31 years of age. All health workers except 1 were female and 16 (64%) were married. Seventeen (68%) of the health workers had 4 or more years of working experience. Thirteen (52%) of the health workers were working in Kilte Awlaelo district; the remaining 12 (48%) in Hintalo Wajerat district.

All health workers had mobile phones prior to enrolment in our study, though none had Android (Google Inc., Mountain View, CA, USA) as an operating system, touch screen interface or local language scripts enabled. Only 3 (12%) of the health workers had ever taken training on basic computer skills though practice did not continue thereafter.

### Health workers’ experiences in using mobile health forms and smartphones

Twenty-three (92%) health workers had completed and submitted at least 3 electronic records to the central database server within the 6-month period of the study. A total of 2,893 electronic health records pertaining to 1,122 women were submitted over the 6-month period. Of this, 1122 were registration records of each woman entered into our system. The remaining 1771 records comprised ANC (782, 44.2%), delivery (491, 27.7%), PNC (237, 13.4%), and ANC laboratory tests (261, 14.7%).

According to the 2011 Ethiopian Demography and Health Survey (EDHS), pregnant women represented approximately 3.8% of the total Ethiopian population [11]. Using this calculation, we expected a total of 1,900 pregnant women to visit the health facilities seeking ANC, delivery or PNC services in our 6-month study period. The 1,122 women entered into our database system using electronic forms on smartphones represented more than half (59.1%) of the expected number of pregnant women in the study area for the study period. We tried to compare the extent to which the electronic forms were utilized by the health workers for pregnant women with the number of pregnant women recorded in paper forms at respective health facilities; however, this was found to be impossible as some facilities did not record the dates at which women visited their facility. Thus, it was difficult to ascertain the accurate number of women visited for a given maternal health care service within a given month.

The distribution of the records submitted to our database system by district showed that an almost equal number of records were submitted from both districts; 897 (56.6%) records from Hintalo Wajerat district, and 874 (49.4%) records from Kilte Awlaelo district. With regards to the distribution of the records submitted by the profession or type of health facility, almost three quarters of the records (1,305, 73.7%), were submitted by midwives (that is from health centres), while the remaining quarter of records (466, 26.3%), were submitted by HEWs (that is from health posts).

Health workers’ use of electronic forms showed a generally consistent trend across the 6 months (Table 1). The first 3 months of the study period saw 689 (38.9%) records submitted. It was encouraging to see the proportion of records submitted in the latter 3 months had increased by 393 (22.2%) to a total 1,082 (61.1%) for that period.

**Table 1:** **Number of records submitted each month by all health workers**

Month	Oct 12	Nov 12	Dec 12	Jan 13	Feb 13	Mar 13	Total
N (/%)	223 (12.6)	283 (16.0)	183 (10.3)	358 (20.2)	436 (24.6)	288 (16.3)	1,771

Over the 6-month period, all health workers used a total of 22,574.18 Ethiopian Birr (ETB) in top-up vouchers, equivalent to 1,254 USD. On average, each health worker had been using a monthly top-up voucher of approximately 150 ETB (8 USD), which showed that an additional top-up voucher of 50 ETB was added from workers’ pockets each month, over and above the monthly 100 ETB provided by us. Of the total amount of voucher used by the health workers over the 6- month period, 20,371.08 ETB (90.2%) were used for voice calls, 2,026.91 ETB (9.0%) for mobile Internet (data) usage and 176.19 ETB (0.8%) for SMS. This expenditure translated, on average, to approximately 163 minutes of voice calls, 29 Mb of Internet data usage and 3 SMSs per health care worker.

The average size of a fully completed electronic record was approximately 2 Kb and the mobile Internet use tariff at the time of the study was 0.046 ETB for 100 Kb. Considering these assumptions, all health workers had used only a sum of only 2.66 ETB (0.13%) of the total mobile top-up voucher for Internet connectivity in submitting records to a central server. The remaining 2,024.25 ETB (99.9%) had been used for other purposes other than submitting completed records. This use of Internet connectivity for other purposes was also evident from the interviews we conducted with the health workers: 10 (43.5%) of whom said they had been using their smartphone for Internet browsing while 6 (26.1%) had been using social media such as Facebook.

### Motivating factors for using mHealth application

Twenty-one (91.3%) of the health workers had been using the smartphone we provided as their primary phone. None supported the idea of leaving a smartphone at a health facility as with other medical equipment; health workers wanted the smartphone to be with them at all times. When we asked why they replaced their private phone with the smartphone as their primary phone, 15 (65.2%) of the health workers said they wanted to use electronic forms and smartphones everywhere and anytime for work and personal purposes, while 14 (60.9%) did not want to carry 2 phones and hence chose to use only the smartphone. All workers believed unrestricted use of the smartphones helped them adapt to the smartphones and electronic forms for work purposes.

Health workers perceived the electronic forms as helpful in several aspects. Twenty (87.0%) workers believed electronic forms and the scorecard were helpful and useful for patient follow-up and keeping the patients' appointments, and 16 (69.6%) workers believed they were good reminders on what to do and what questions needed to be asked. Twelve (52.2%) workers said electronic forms were comprehensive and 10 (43.5%) workers said they were helpful to ask questions and assess patients step-by-step. Further, 9 (39.1%) workers perceived electronic forms as learning tools, and 6 (26.1%) workers perceived they could be used everywhere and anytime.

### Barriers for using electronic forms and smartphones

Over the 6 months, no pregnant woman declined a health worker to use the electronic forms on smartphone for assessment and interview. No health worker felt any problem interacting with women when they used the smartphone and electronic forms for interview and assessment. Twenty-one of the 23 health workers found using the smartphone and electronic forms for data collection and patient assessment to be much easier. They found the touch and size screen of the smartphone and keyboards were easy to get used to, and all except one said the mobile network connectivity in their respective village was consistently good enough for record submission. All health workers found the mobile scorecard very helpful for their work. However, when asked if they had used the smartphones and electronic forms for assessing all women coming to their health facility, all workers except one said they did not interview all women using the electronic forms and smartphone.

The barriers for not using electronic forms consistently mainly stemmed from the health system, health workers’ behaviour, and the workflow we followed in implementing this study (Table 2). In this study, we required the health workers to fill out both the existing paper forms at health facility and the electronic forms simultaneously. This was considered as time consuming and a major reason for not using the electronic forms all the time as mentioned by 18 (78.3%) of the health workers. With regards to health workers’ behaviour, most of the health workers had been travelling away from their working station for different reasons. For instance, within the 6-month period of the study, 19 (82.6%) health workers had been away from their health facility at least once for attending training outside of their working station. Table 2 shows barriers that health workers encountered in using the electronic forms and smartphone at least once during the study period.

**Table 2:** **Barriers for using electronic forms and smartphones by health extension workers and midwives (N = 23)**

Reasons related	Reasons	Frequency number (%)
Electronic forms, application and smartphone	Electronic forms were vast and take a long time to complete	11 (47.8%)
	Problem with user name and password setting	5 (21.7%)
	Electronic forms had required questions which cannot be skipped; for example, LMP	4 (17.4%)
	Smartphone froze or locked up	9 (39.1%)
	Smartphone’s battery ran out of charge	5 (21.7%)
	Smartphone had insensitive screen or keys	3 (13.0%)
Health workers’ behaviour	Ran out of mobile top-up balance	10 (43.5%)
	Health worker changed smartphone’s date and time setting (Julian and Gregorian calendar confusion)	9 (39.1%)
	Accidentally deleted installed electronic forms and/or ODK application from smartphone	7 (30.4%)
	Health workers’ reluctance or negligence to use electronic forms	5 (21.7%)
	Accidentally inactivated smartphone’s GPRS network	4 (17.4%)
	Lost the smartphone	1 (4.3%)
Health system	Health workers were not at their working place for attending training somewhere out of their working place	19 (82.6%)
	Health workers had to enter the data of a woman in two or more forms (electronic, paper and other), which was time consuming	18 (78.3%)
	Workload and high number of patient flow	15 (65.2%)
	Health workers were not at their health facility for social reasons such as wedding, mourning or funeral	9 (39.1%)
	Heath workers had annual leave	7 (30.4%)
	Priority was for filling out paper forms over electronic forms	7 (30.4%)
	Main focus was for recording ANC and negligence on keeping delivery and PNC records	2 (8.7%)

### Health workers’ preferences and intention to use electronic forms

If paper forms were to be replaced by electronic forms in the future, all health workers expressed their intention to use electronic forms without any reservation. If they were given a chance to choose paper form or electronic form, 22 (95.7%) said they would have chosen electronic forms over paper forms. With regards to language preference, 18 (78.3%) said they preferred to use the local language (Tigrinya) version of the forms as it was easier for them to understand and communicate with women. Five (21.7%) of the health workers who preferred the English version of the forms were midwives whose reasons were that medical terms were more easily understood in English than in the local language.

## Discussion

Over the 6-month period, health workers used the electronic forms in a total of 1122 women and the overall use of the forms across the study period was virtually consistent. Almost three quarters of the records were submitted by midwives while the remaining quarter of records were submitted by HEWs. All health workers preferred unrestricted use on the smartphone and its functions. Our analysis showed health workers used 90.2% of their mobile top-up voucher for making voice calls.

The mHealth framework for Ethiopia, issued in 2011 by the Ethiopian Federal Ministry of Health and its partners, only deals with the needs and opportunities of mobile technologies for HEWs [20]. Other frameworks and white papers for mHealth in developing countries mainly focus on the use of mHealth for community health workers [16–19]. However, recent studies on maternal health service utilization in Ethiopia showed that the proportion of women who are seeking and getting maternal health care at health centres from midwives and nurses is increasing [6, 8, 27]. These studies noted that some rural women are receiving maternal health care at health centres, bypassing the HEWs [6, 8]. Most importantly, this study showed midwives found the electronic forms on smartphones equally as useful as the HEWs. Hence, to exploit the potential benefits of mHealth applications in strengthening and facilitating data exchange and referrals in relation to maternal health care at primary health care, it would be beneficial to also consider the mHealth needs of midwives and other midlevel health professionals at health centres.

Our observation that HEWs and midwives assessed and electronically entered into our system a large number of pregnant women despite having to use both the electronic forms and existing paper forms is an encouraging finding suggesting that electronic forms on smart phones may indeed be feasible at scale. Nevertheless, the barriers identified in this study call for implementers of mHealth initiatives to be cautious and take the necessary precautions before implementing such interface at scale. Unforeseen challenges such as 21% of problems with username and password setting and 30% of accidently deleted installed electronic forms (Table 2), might be manageable and seem insignificant in a pilot study. However, these challenges may severely limit thousands of workers’ productivity when implementing such interface at scale. Hence, implementers of such interface should make sure that there are trained technical persons who can solve such problems and provide support to health workers. Besides, certifying health workers during training whether they are able to use the application appropriately or not and supporting health workers with a brief and guiding manual or brochure on how to use the application and forms appropriately might be helpful in minimizing such technical barriers.

Another challenge in implementing electronic forms on smartphone at scale is covering the cost of airtime. Although there is no clear evidence, electronic forms could potentially be useful to minimize costs of using paper forms [26, 28]. The decision on whether to put restrictions on the use of smartphones and Internet connectivity when employing smartphone-based electronic forms may affect the operational cost of implementing such an interface. In this study, we did not put any restriction on the use of the smartphone’s function, Internet connectivity and mobile top-up. We found that unrestricted use of the smartphone helped and motivated health workers to get used to the electronic forms. Despite this noted benefit of unrestricted use of smartphones, our analysis on health workers’ use of mobile top-up voucher implies the need of limiting health workers’ airtime use for only intended purposes on the basis of costs. In this study, health workers used about 90% of their mobile top-up for voice calls. On average, each health worker had made approximately 163 minutes of voice calls every month. Additionally, as health workers become handier with their smartphone, their use of Internet connectivity through their smartphone for other purposes such as Internet browsing and Facebook will increase. Though this may help health workers to independently gain access to information and other resources on the Internet, it will compromise the primary purpose of using electronic forms for patient assessment, and incur additional costs to the health system. Thus, it would be necessary to manage and restrict health workers’ airtime use. Covering such costs in a larger-scale implementation of similar projects for a longer period may be difficult and unfeasible. Hence, implementers of such an interface should solicit a mechanism to provide health workers free airtime for uploading forms or restrict the use of top-up vouchers for only the required purpose.

This study was done among a small number of health workers. As a result it becomes difficult for the study to analyze health workers’ experiences, barriers, preferences and motivating factors in using electronic forms on smartphones by sociodemographic characteristics and other factors. This study gives a glimpse of the bigger picture. Hence, we recommend larger studies to investigate individual factors that affect health workers’ use of mobile health forms. Well designed qualitative studies might also be helpful to dig out health system and health workers’ behaviour-related factors.

With the good intention of investigating and understanding health workers’ experiences in using electronic forms on smartphones, two of the investigators conducted the data collection and analysis of the study. However this might have lead to information bias. To minimize this bias, we pretested the questionnaire and discussed the findings and arguments made in this study among all research team members who have different areas of expertise.

## Conclusion

Both HEWs and midwives found electronic forms on smartphones useful for day-to-day maternal health care services delivery. However, technical challenges related to design of electronic forms and smartphones, problems of health systems and health workers’ behaviour would make using such forms on smartphones at scale difficult. Furthermore, soliciting a mechanism of securing free airtime for health workers from telecommunication service providers, or putting restrictions on health workers’ mobile top-up voucher use would be necessary in view of long-term cost management and sustainable implementation of such work tools.

## Electronic supplementary material

Additional file 1: **Checklist/questionnaire for assessment of health workers’ usability of mHealth forms.** (DOC 150 KB)

## Abbreviations

ANC: Antenatal Care
CPR: Contraceptive Prevalence Rate
EDHS: Ethiopian Demography and Health Survey
ETB: Ethiopian Birr
GPRS: General Packet Radio Service
GPS: Global Positioning System
HEP: Health Extension Programme
HEWs: Health Extension Workers
LMP: Last Menstrual Period
mHealth: Mobile Health
ODK: Open Data Kit
PDAs: Personal Digital Assistants
PHCUs: Primary Health Care Units
PNC: Postnatal Care
SMS: Short Message Services
WHO: World Health Organization.
