# Supplementary material for: Health workers’ experiences, barriers, preferences and motivating factors in using mHealth forms in Ethiopia
Source: Hum Resour Health. 2015 Jan 15;13(1):2. doi: 10.1186/1478-4491-13-2 (PMC4325949; doi:10.1186/1478-4491-13-2)
Supplement: Supplementary file 1 — Additional file 1: Checklist/questionnaire for assessment of health workers’ usability of mHealth forms. (DOC 150 KB) [file 12960_2014_470_MOESM1_ESM.doc]

Checklist for health worker’s usability

This checklist is prepared to collect data on the usability of mHealth application and electronic forms by health workers.

**Checklist for health workers’ usability**

| Section 1- Health facility data on maternal health utilization for comparison with data on overall utilization of smartphone based electronic form. | | | |
| --- | --- | --- | --- |
| S.no | Question | Response | Remark |
|  | Usability by Health worker |  |  |
| 101 | Name of the health worker |  |  |
| 102 | Name of the health center/health post |  |  |
| 103 | How many women have you visited for ANC from Meskerem 1, 2005- Miazia 30,2005**?** ( number of women) | Paper _________ |  |
| 104 | Please put the number of ANC visitors (paper report) by month | Meskerem _________  Tikmiti___________  Hidar ____________  Tahsas_______________  Tiri_____________  Yekatit_____________  Megabit_____________  Miazia______________ |  |
| 105 | How many women you have visited for ANC from Meskerem1 2005- Miazia 30, 2005 had at least one ANC lab test? ( number of women) | Paper _________ |  |
| 106 | Please put the number of women who had at least one ANC lab test (paper report) by month | Meskerem _________  Tikmiti___________  Hidar ____________  Tahsas_______________  Tiri_____________  Yekatit_____________  Megabit_____________  Miazia______________ |  |
| 107 | How many women have you assisted for birth from meskerem 1,2005 – Miazia 30, 2005? ( number of women) | Paper _________ |  |
| 108 | Please put the number of women you have assisted for birth ( paper report) by month | Meskerem _________  Tikmiti___________  Hidar ____________  Tahsas_______________  Tiri_____________  Yekatit_____________  Megabit_____________  Miazia______________ |  |
| 109 | How many women have you visited for PNC from Meskerem 1, 2005 – Miazia 30, 2005? ( number of women) | Paper _________ |  |
| 110 | Please put the number of PNC visitors ( paper report) by month | Meskerem _________  Tikmiti___________  Hidar ____________  Tahsas_______________  Tiri_____________  Yekatit_____________  Megabit_____________  Miazia______________ |  |
| 111 | How have you been usually filling out the electronic form? | Before completing paper at the time of interviewing woman  After paper form but still when the mother is in the room  After paper and when the mother already left the room  Both at the same time |  |
| 112 | If you were usually completing the electronic form before the paper form when the mother is still in the room, what is the reason for doing so? |  |  |
| 113 | If you were usually completing the electronic form after the paper form when the mother is still in the room, what is the reason for doing so? |  |  |
| 114 | If you were usually completing the electronic form some time after the paper form when the mother is already gone, what is the reason for doing so? |  |  |
| 115 | If you were usually completing the electronic and paper forms at the same time, what is the reason for doing so? |  |  |
| 116 | We observed there are differences in the number of women recorded in paper forms and in the number of women whose records is submitted to our server using the smartphone, * usually less for electronic forms, would you please tell us why this happened? |  |  |
|  |  |  |  |
| **Section 2 – Factors affecting usability:** | | | |
| 201 | At this time, do you use mobile phone do you use as primary smartphone? The smartphone we gave you or your mobile phone? | The smartphone  My mobile phone  Both |  |
| 201A | If you use the smartphone as your primary phone, why? |  |  |
| 202 | What were the conditions or situations that motivated you to use the smartphone and electronic forms in your day to day health care provision? Would you please tell us in details? |  |  |
| 203 | What were the conditions or situations that prohibited or discourage you in using the smartphone and electronic forms in your day to day health care provision? Would you please tell us in details? |  |  |
| 204 | Since you started working with us and using smartphone for interviewing women did you interviewed all women coming to your HP/HC using the smartphone and electronic form we gave you? ( please electronic report vs paper report) | Yes  No |  |
|  | Ask the health worker whether or not since he/she has started using the electronic forms and smartphone for submitting real patient record encountered the following conditions: |  |  |
| 204A | Did you accidentally deleted or removed forms or ODK from your smartphone? | Yes  No |  |
| 204A1 | If yes, did this stop or interrupt you from submitting real patient record? | Yes  No |  |
| 204A2 | If yes, why or how? |  |  |
| 204B | Did your username and password stop working? | Yes  No |  |
| 204B1 | If yes, did this stop or interrupt you from submitting real patient record? | Yes  No |  |
| 204B2 | If yes, why or how? |  |  |
| 204C | Did you have a problem with date and time setting? | Yes  No |  |
| 201C1 | If yes, did this stop or interrupt you from submitting real patient record? |  |  |
| 204C2 | If yes, why or how? |  |  |
| 204D | Did you have insensitive screen? | Yes  No |  |
| 204D1 | If yes, did this stop or interrupt you from submitting real patient record? | Yes  No |  |
| 204D2 | If yes, why or how? |  |  |
| 204E | Did you have insensitive keys? | Yes  No |  |
| 204E1 | If yes, did this stop or interrupt you from submitting real patient record? | Yes  No |  |
| 204E2 | If yes, why or how? |  |  |
| 204F | Did the batteries of your smartphone run out of charge when you were interviewing or wanted to use your smartphone for interview? | Yes  No |  |
| 204F1 | If yes, did this stop or interrupt you from submitting real patient record? | Yes  No |  |
| 204F2 | If yes, why or how? |  |  |
| 204G | Did someone called you in the middle of your interview? | Yes  No |  |
| 204G1 | If yes, did this stop or interrupt you from submitting real patient record? | Yes  No |  |
| 204G2 | If yes, why or how? |  |  |
| 204H | Did your smartphone get freeze or stack? | Yes  No |  |
| 204H1 | If yes, did this stop or interrupt you from submitting real patient record? |  |  |
| 204H2 | If yes, why or how? |  |  |
| 204I | Did your Amharic keyboard removed accidently from your smartphone? | Yes  No |  |
| 204I1 | If yes, did this stop or interrupt you from submitting real patient record? |  |  |
| 204I2 | If yes, why or how? |  |  |
| 204J | Did you have difficulty in switching the keyboard from Amharic to English and vice versa? | Yes  No |  |
| 204J1 | If yes, did this stop or interrupt you from submitting real patient record? | Yes  No |  |
| 204J2 | If yes, why/how? |  |  |
| 204K | Did you accidentally remove your memory card from your smartphone? | Yes  No |  |
| 204K1 | If yes, did this stop or interrupt you from submitting real patient record? | Yes  No |  |
| 204K2 | If yes, why/how? |  |  |
| 204L | Did you accidentally save music and leave no space for saving completed records? | Yes  No |  |
| 20141 | If yes, did this stop or interrupt you from submitting real patient record? | Yes  No |  |
| 20142 | If yes, why/how? |  |  |
| 204M | Did you forget your smartphone carrying with you all the time when you are at work? | Yes  No |  |
| 204M1 | If yes, did this stop or interrupt you from submitting real patient record? | Yes  No |  |
| 204M2 | If yes, why/how? |  |  |
| 204N | Did you run out of balance in a month? | Yes  No |  |
| 204N1 | If yes, did this stop or interrupt you from submitting real patient record? | Yes  No |  |
| 204N2 | If yes, why/how? |  |  |
| 204O | Did you forget your smartphone carrying with you all the time when you are at work? | Yes  No |  |
| 204O1 | If yes, did this stop or interrupt you from submitting real patient record? | Yes  No |  |
| 204O2 | If yes, why/how? |  |  |
| 204P | Did you have to enter and complete patient information in more than one recording format? | Yes  No |  |
| 204P1 | If yes, did this stop or interrupt you from submitting real patient record? | Yes  No |  |
| 204P2 | If yes, why/how? |  |  |
| 204Q | When you compare to your paper recording, did it take longer time to fill an electronic form * e.g. ANC form? | Yes  No |  |
| 204Q1 | If yes, did this stop or interrupt you from submitting real patient record? | Yes  No |  |
| 204Q2 | If yes, why/how? |  |  |
| 204R | Did you have annual leave? | Yes  No |  |
| 204R1 | If yes, did this stop or interrupt you from submitting real patient record? | Yes  No |  |
| 204R2 | If yes, why/how? |  |  |
| 204S | Did you go out of your working kebele for some days for training? | Yes  No |  |
| 204S1 | If yes, did this stop or interrupt you from submitting real patient record? | Yes  No |  |
| 204S2 | If yes, why/how? |  |  |
| 204T | Did you have other social reasons * such as wedding, mourning and funeral? | Yes  No |  |
| 204T1 | If yes, did this stop or interrupt you from submitting real patient record? | Yes  No |  |
| 204T2 | If yes, why/how? |  |  |
| 204V | Did you have other problems related with your work, supervisors, and district health office? | Yes  No |  |
| 204V1 | If you had, would you please tell us the problems? |  |  |
| 204V2 | If yes, did this stop or interrupt you from submitting real patient record? | Yes  No |  |
| 204V3 | If yes, why/how? |  |  |
| 204W | Were there other conditions that prevented from you using the smartphone and electronic forms other than the factors mentioned above? | Yes  No |  |
| 204W1 | If yes, would you please tell us? |  |  |
| 204X | Do you think the smartphone should be kept at the HC or HP all the time? | Yes  No |  |
| 204X1 | If yes, why? |  |  |
| 204X2 | If no, why? |  |  |
| 204Y | Do you think the unrestricted use of the smartphone helped you to use practice and use the smartphone well? | Yes  No |  |
| 204Y1 | If yes, why? |  |  |
| 204Y2 | If no, why? |  |  |
| Section 3/ easiness / satisfaction questions tell me your responses by rating 1- 5 | | | |
| Number | Question | Rate | Reason |
| 301 | How did you find the GPRS connectivity in your area for submitting completed form? | 1. Very poor 2. Poor 3. Neutral 4. Good 5. Very good |  |
| 302 | How did you find the lifetime of the batteries of the smartphones for using in your daily work? | 1. Very inadequate 2. Inadequate 3. Average 4. Adequate 5. Very adequate |  |
| 303 | How did you find the easiness of the HTC android device or keyboard in entering text fields (data) in English? | 1. Much harder 2. Harder 3. Neutral 4. Easier 5. Much easier |  |
| 304 | How did you find the easiness of the HTC android device or keyboard in entering text fields (data) in Tigrigna? | 1. Much harder 2. Harder 3. Neutral 4. Easier 5. Much easier |  |
| 305 | How did you find the easiness of the HTC android device or keyboard in entering numeric data? | 1. Much harder 2. Harder 3. Neutral 4. Easier 5. Much easier |  |
| 306 | How did you find the easiness of the HTC android device and electronic devices in collecting data? | 1. Much harder 2. Harder 3. Neutral 4. Easier 5. Much easier |  |
| 307 | How did you find the time taken to complete a form using the HTC android device? Was it faster or slower? | 1. Much slower 2. Slower 3. Neutral 4. Faster 5. Much faster |  |
| 308 | Does the HTC android device make it easier or harder to interact with individuals? | 1. Much harder 2. Harder 3. Neutral 4. Easier 5. Much easier |  |
| 309 | How did you find the screen size of the smartphone for collecting patient record or data? | 1. Much harder 2. Harder 3. Neutral 4. Easier 5. Much easier |  |
| 310 | How did you find the touch screen of the smartphone for collecting patient record or data? | 1. Much harder 2. Harder 3. Neutral 4. Easier 5. Much easier |  |
| 311 | How often o you login into the scorecard? | 1. Not at all 2. Rarely 3. Often 4. Usually 5. Always |  |
| 312 | How did you find the scorecard helpful for your work? | 1. Not at all 2. Rarely 3. Often 4. Usually 5. Always |  |
| 313 | Should end-users continue to use android devices and electronic forms? | 1. Definitely discontinue 2. Discontinue 3. Neutral 4. Continue 5. Definitely continue |  |
| 314 | Was your training on using the android device good or bad? | 1. Much poor 2. Poor 3. Neutral 4. Good 5. Very good |  |
| Section 4/ preference questions | | | |
| 401 | In general, which do you prefer to use the electronic form or paper form? | Paper form  Electronic form |  |
| 401A | If paper form, why? |  |  |
| 401B | If electronic form, why? |  |  |
| 402 | Which do you prefer, the English version or Tigrigna version of the electronic forms? | English  Tigrigna  Both  No difference |  |
| 401A | If paper form, why? |  |  |
| 401B | If electronic form, why? |  |  |
| Section 5- other applications | | | |
| Number | Question | Rate | Reason |
| 502 | Do you use the smartphone for other purposes other than submitting data? Like facebook and other purposes | Yes  No |  |
| 503 | If yes, for what purpose? |  |  |
| 504 | Finally what do you suggest to scale up such kind of mobile phone based technology and application? |  |  |
